# Association between triglyceride glucose-body mass index and asthma among US participants with type 2 diabetes: An observational study

**DOI:** 10.1097/MD.0000000000046260

**Published:** 2025-11-28

**Authors:** Shengjia Zheng, Minghe Wang, Yi Fang, Yuanmei Gao

**Affiliations:** a Department of Respiratory and Critical Care Medicine, Guangdong Provincial Key Laboratory of Major Obstetric Diseases, Guangdong Provincial Clinical Research Center for Obstetrics and Gynecology, The Third Affiliated Hospital, Guangzhou Medical University, Guangzhou, China.

**Keywords:** asthma, cross-sectional study, insulin resistance, NHANES, TyG-BMI, type 2 diabetes

## Abstract

This study explores the association between triglyceride glucose-body mass index (TyG-BMI) and asthma in US individuals with type 2 diabetes (T2D) using data from the National Health and Nutrition Examination Survey 2005 to 2016. The participants included 3236 adults aged >20 years with type 2 diabetes. The primary outcome measure was the association between TyG-BMI and asthma prevalence, and the secondary outcome measure was the threshold effect analysis and subgroup analyses. A significant nonlinear relationship between TyG-BMI and asthma risk was found (nonlinearity *P* = .016). When TyG-BMI exceeded 208.96, each 10-unit increase was linked to a 5.4% higher asthma risk (OR = 1.054, 95% CI 1.034–1.074, *P* < .001). Subgroup analyses showed this relationship was consistent across different age groups, genders, races, and family asthma histories, especially in individuals aged 65 and older with T2D. In US individuals with T2D, there is a significant nonlinear association between TyG-BMI and asthma risk. TyG-BMI can be an important indicator for assessing asthma risk in this group, and metabolic control is crucial for asthma prevention and management.

## 1. Introduction

Asthma, which is marked by bronchial inflammation and reversible airflow obstruction, presents a major worldwide health challenge for affected individuals.^[1]^ This chronic condition is defined mainly by recurrent episodes of cough, wheeze, and dyspnea, arising from airway inflammation and hyperresponsiveness.^[2]^ Asthma is a serious global health issue, affecting an estimated 235 million people worldwide,^[3]^ including approximately 24 million in the United States. A rising trend in asthma prevalence has been observed, with the condition impacting 3.1% of the US population in 1980 and subsequently affecting 8.3% of the population by 2016.^[4]^ Thus, identifying intervenable risk factors is crucial for mitigating this burden.

The comorbidity of asthma and type 2 diabetes (T2D) leads to worse outcomes and a heavy global burden, compounded by poorer quality of life.^[5]^ T2D-associated asthma severity promotes systemic corticosteroid use, which exacerbates glycemic dysfunction.^[6]^ Research shows that these 2 diseases have complex interactions, which may involve joint effects of inflammation, obesity and insulin resistance (IR).^[5]^ The development of T2D is primarily associated with low-grade chronic inflammation-induced insulin resistance, notably elevated levels of inflammatory mediators such as high-sensitivity C-reactive protein, tumor necrosis factor, and interleukin-6 (IL-6), which are significantly correlated with an increased risk of diabetes.^[7]^ Similarly, the core pathogenesis of asthma involves excessive activation of the nuclear factor kappa B signaling pathway, which drives key pathological processes including allergic airway inflammation and mucus hypersecretion.^[8]^ The convergence of these inflammatory mechanisms suggests shared pathophysiological pathways, potentially explaining their frequent co-occurrence. Obesity, a chronic condition characterized by an underlying low-grade inflammatory state, may interact with this inflammation to enhance the development of diabetes,^[9]^ research have indicated that obesity and IR, both mediated by low-grade systemic inflammation, are key drivers of asthma pathogenesis.^[10]^

Additionally, the relationship between IR and asthma has garnered widespread attention. IR precedes hyperglycemia and leads to compensatory hyperinsulinemia.^[11]^ It has demonstrated an association with asthma in individuals with diabetes and is proposed to have a causal role, as indicated by animal studies.^[12]^ In an animal study, insulin-treated mice demonstrated peribronchial thickening, collagen deposition, and increased airway hyperresponsiveness, similar to the pathological changes seen in asthma.^[13]^ Hyperinsulinemia promotes nonallergic airway remodeling in asthma by excessively activating the insulin/IGF-1 signaling pathway, stimulating fibroblast proliferation and differentiation, and leading to collagen deposition and fibrosis.^[14]^

The homeostasis model assessment of insulin resistance (HOMA-IR) index and triglyceride-glucose index (TyG index) can reflect the state of IR associated with various metabolic diseases.^[15]^ As one of the assessment tools for IR, HOMA-IR has limitations in accuracy and reproducibility.^[16]^ Triglyceride glucose-body mass index (TyG-BMI), derived from the TyG index, integrates triglycerides (TG), fasting blood glucose, and body mass index (BMI) to provide enhanced IR detection.^[17]^

Despite the recognition of a link between metabolic indices and respiratory diseases, current research has not adequately explored the relationship between TyG-BMI and asthma, particularly within the context of T2D in the United States. This knowledge gap warrants additional research aimed at uncovering potential underlying mechanisms and guiding targeted interventions. The present study utilizes data from the National Health and Nutrition Examination Survey (NHANES) to address this deficit, offering novel insights into the association between TyG-BMI and asthma in individuals with T2D.

## 2. Methods

### 2.1. Study population

The NHANES are representative cross-sectional surveys designed by the National Center for Health Statistics (NCHS).^[18]^ Approval for the NHANES study protocols was granted by the NCHS Institutional Review Board, with informed consent obtained from all participants prior to their involvement. In accordance with National Institutes of Health policy, such analysis involving de-identified data that was not directly in contact with participants was not considered human subjects study and was not subject to institutional review board review. The NCHS Ethics Review Board approved all NHANES protocols, as this study exclusively utilized de-identified, publicly available data, it qualified for exemption from additional institutional review board approval. Patients or the public were not involved in the design, conduct, reporting, or dissemination plans of our research. Further information regarding the NHANES data is available on the CDC website (https://www.cdc.gov/nchs/nhanes/index.html).

**Figure 1.**
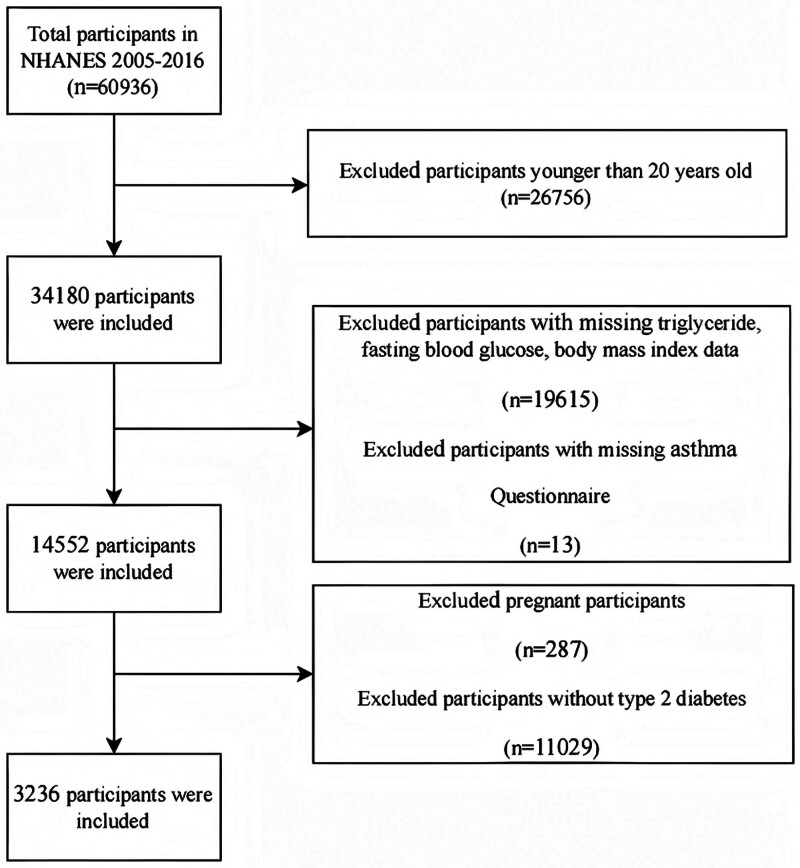
Flowchart for inclusion of study participants.

We acquired data of participants with T2D from NHANES 2005 to 2016. T2D was defined by the American Diabetes Association criteria^[19]^ and a self-report questionnaire. Participants who fulfilled the following criteria were identified as T2D^[20]^: glycated hemoglobin ≥ 6.5%, fasting plasma glucose ≥ 7 mmol/L; during an oral glucose tolerance test, 2-hour plasma glucose ≥ 11.1 mmol/L; self-report questionnaire data indicating physician diagnosis of diabetes; and lower blood glucose by current use of insulin or diabetes pill. We excluded participants with missing information of TG, fasting blood glucose, body mass index (BMI) and asthma questionnaire. Finally, 3236 participants were analyzed (Fig. 1).

### 2.2. Assessment of asthma

To evaluate the medical conditions of the participants, a questionnaire-based survey was conducted. Specifically, the participants were queried as to whether they had ever received a diagnosis of asthma from a doctor or another healthcare professional. Those who responded in the affirmative were categorized as having asthma, whereas the ones who responded negatively were considered as not having asthma.

### 2.3. Assessment of TyG-BMI index

TyG-BMI was calculated as follows: BMI = weight (kg)/height (m^2^); TyG index = Ln[1/2 fasting glucose (mg/dL) × fasting TGs (mg/dL)]; TyG-BMI = TyG index × BMI.

### 2.4. Assessment of covariates

Covariates were chosen based on prior research and theoretical reasoning. Information on age, sex, race, education level, marital status, poverty income ratio (PIR), smoking status, alcohol consumption, hypertension status, family history of diabetes, family history of asthma, and biochemical indicators, including high-density lipoprotein cholesterol (HDL), low-density lipoprotein cholesterol (LDL), total cholesterol (TC), aspartate aminotransferase, alanine aminotransferase (ALT), eosinophil count, eosinophil percentage, neutrophil count, and neutrophil percentage, was ascertained through standardized questionnaires and laboratory tests.

Race was categorized as non-Hispanic white, non-Hispanic black, Mexican American, or other races. Education level was categorized into 3 levels based on the number of years of education: <9 years, 9 to 12 years, and >12 years. Participants were divided into 2 groups according to marital status: living alone (never married, separated, divorced, and widowed) and living with a partner (married and living with partner). Family income was divided into 3 levels according to the PIR: low income (PIR ≤ 1.3), medium income (PIR = 1.3–3.5), and high income (PIR > 3.5). Smoking status was categorized as never smokers (smoked <100 cigarettes), current smokers, and former smokers (quit smoking after smoking >100 cigarettes). Participants were categorized according to alcohol drinking status as never (<12 drinks in their lifetime), former (≥12 drinks in 1 year and did not drink last year, or did not drink last year but drank ≥ 12 drinks in their lifetime), or current drinkers (≥12 drinks in any 1 year and did drink last year). Family history of diabetes was derived from a question: “Had any of your biological relatives been diagnosed with diabetes by a healthcare professional?” Family history of asthma was derived from a question: “Had any of your biological relatives been diagnosed with asthma by a healthcare professional?”

The height, weight, and blood pressure were measured in accordance with standardized protocols. BMI was calculated by dividing weight in kilograms by height in meters squared (kg/m^2^). According to the 2017 American College of Cardiology/American Heart Association hypertension guidelines, hypertension was defined as being currently taking antihypertensive drugs, or if not, having systolic blood pressure level ≥ 130 mm Hg and/or diastolic blood pressure level ≥ 80 mm Hg.^[21]^

### 2.5. Statistical analysis

Data are presented as mean ± standard deviation for normally distributed continuous variables, median (interquartile range) for non-normally distributed continuous variables, and frequency or percentage for categorical variables. Continuous variables were analyzed using the Kruskal–Wallis test (nonparametric) or one-way analysis of variance (parametric), and categorical variables were assessed with the Chi-squared test. A univariable logistic regression analysis was conducted to investigate associations between independent covariates and asthma, with results presented in Table 2. Following this, a multivariable logistic regression analysis with model adjustment was performed in Table 3. We used TyG-BMI as a continuous and categorical variable (quartiles: Q1 [<234.705]; Q2 [234.705–<277.245]; Q3 [277.245–<328.279]; and Q4 [≥328.279]) to analyze its relationship with asthma. Crude model was unadjusted. Model 1 was adjusted for age, gender, and race. Model 2 was adjusted for the variables in Model 1 plus marital status, education level, and PIR. Model 3 was adjusted for the variables in Model 2 plus smoking status, drinking status, hypertension, family history of diabetes, family history of asthma. Model 4 was adjusted for the variables in Model 3 plus HDL, LDL, ALT, aspartate aminotransferase, TC, eosinophil count, percentage of eosinophils, neutrophil count, neutrophil percentage. Subgroup analysis was used to examine the relationship between TyG-BMI and asthma according to the age, sex, race, family history of asthma. In addition, we examined the linear relationship between TyG-BMI and asthma using restricted cubic splines, and using a 2-piecewise logistic regression model to examine the threshold association between TyG-BMI and asthma.

**Table 1:** Characteristics of study participants.

Characteristics	Overall	TyG-BMI quartiles				*P*
		Q1 (<234.705)	Q2 (234.705–<277.245)	Q3 (277.245–<328.279)	Q4 (≥328.279)	
Sample size, n (%)	3236 (100)	809 (25)	809 (25)	809 (25)	809 (25)	
Age(years)	60.4 ± 14.6	62.3 ± 16.0	62.9 ± 13.9	60.6 ± 13.5	55.6 ± 13.6	<.001
**Sex, n (%**)						<.001
Female	1566 (48.4)	356 (44)	347 (42.9)	415 (51.3)	448 (55.4)	
Male	1670 (51.6)	453 (56)	462 (57.1)	394 (48.7)	361 (44.6)	
**Race, n (%**)						<.001
Non-Hispanic white	1276 (39.4)	341 (42.2)	301 (37.2)	305 (37.7)	329 (40.7)	
Non-Hispanic black	759 (23.5)	164 (20.3)	195 (24.1)	192 (23.7)	208 (25.7)	
Mexican American	576 (17.8)	101 (12.5)	144 (17.8)	177 (21.9)	154 (19)	
Other races	625 (19.3)	203 (25.1)	169 (20.9)	135 (16.7)	118 (14.6)	
**Education level, n (%**)						.004
<9 years	574 (17.7)	137 (16.9)	148 (18.3)	177 (21.9)	112 (13.8)	
9–12 years	1332 (41.2)	333 (41.2)	341 (42.2)	310 (38.3)	348 (43)	
>12 years	1330 (41.1)	339 (41.9)	320 (39.6)	322 (39.8)	349 (43.1)	
**Marital status, n (%**)						.596
Living with partner	1952 (60.3)	490 (60.6)	497 (61.4)	493 (60.9)	472 (58.3)	
Living alone	1284 (39.7)	319 (39.4)	312 (38.6)	316 (39.1)	337 (41.7)	
**PIR, n (%**)						.362
≤1.3	1028 (35.0)	235 (32.5)	253 (34)	258 (35.3)	282 (38.1)	
1.3–3.5	1191 (40.6)	308 (42.7)	311 (41.8)	285 (39)	287 (38.8)	
>3.5	717 (24.4)	179 (24.8)	180 (24.2)	187 (25.6)	171 (23.1)	
**Smoke, n (%**)						.105
Never smoking	1632 (50.5)	418 (51.7)	401 (49.6)	402 (49.8)	411 (50.9)	
Former smoker	1038 (32.1)	230 (28.5)	270 (33.4)	281 (34.8)	257 (31.8)	
Current smoker	563 (17.4)	160 (19.8)	138 (17.1)	125 (15.5)	140 (17.3)	
**Drinking, n (%**)						.29
Never drinking	546 (18.3)	130 (17.8)	133 (18)	136 (18.1)	147 (19.1)	
Former drinker	507 (17.0)	109 (14.9)	119 (16.1)	130 (17.3)	149 (19.4)	
Current drinker	1938 (64.8)	493 (67.3)	486 (65.9)	485 (64.6)	474 (61.6)	
**Hypertension, n (%**)						<.001
Yes	1668 (51.7)	344 (42.7)	394 (48.9)	450 (56)	480 (59.4)	
No	1556 (48.3)	462 (57.3)	412 (51.1)	354 (44)	328 (40.6)	
HDL (mg/dL), mean ± SD	50.5 ± 15.7	58.8 ± 18.6	50.9 ± 14.5	47.2 ± 12.9	45.0 ± 12.4	<.001
LDL (mg/dL), mean ± SD	108.0 ± 37.7	104.4 ± 36.5	109.2 ± 38.5	109.1 ± 38.8	109.2 ± 36.9	.024
TC, mg/dL, mean ± SD	189.4 ± 46.3	183.3 ± 42.7	188.8 ± 46.0	190.9 ± 46.8	194.6 ± 49.1	<.001
ALT (µ/L), median (IQR)	22.0 (17.0, 30.0)	20.0 (16.0, 27.0)	22.0 (17.0, 29.0)	23.0 (17.0, 32.0)	24.0 (18.0, 33.0)	<.001
AST (µ/L), median (IQR)	23.0 (20.0, 28.0)	23.0 (20.0, 28.0)	23.0 (20.0, 28.0)	23.0 (19.0, 28.0)	24.0 (19.5, 30.0)	.315
Eosinophil count, median (IQR)	0.2 (0.1, 0.3)	0.2 (0.1, 0.3)	0.2 (0.1, 0.3)	0.2 (0.1, 0.3)	0.2 (0.1, 0.3)	<.001
Percentage of eosinophils, median (IQR)	2.6 (1.7, 3.8)	2.5 (1.7, 3.9)	2.6 (1.7, 3.7)	2.7 (1.8, 3.9)	2.6 (1.8, 3.8)	.2
Neutrophil count, mean ± SD	4.3 ± 1.7	4.1 ± 1.7	4.2 ± 1.7	4.3 ± 1.6	4.7 ± 1.8	<.001
Percentage of neutrophil, mean ± SD	59.1 ± 9.7	59.2 ± 10.4	59.1 ± 9.9	58.2 ± 9.0	59.8 ± 9.3	.013
**Family history of asthma, n (%**)						.168
Yes	570 (17.6)	135 (16.7)	128 (15.8)	147 (18.2)	160 (19.8)	
No	2666 (82.4)	674 (83.3)	681 (84.2)	662 (81.8)	649 (80.2)	
**Family history of diabetes, n (%**)						<.001
Yes	1824 (56.4)	385 (47.6)	442 (54.6)	489 (60.4)	508 (62.8)	
No	1412 (43.6)	424 (52.4)	367 (45.4)	320 (39.6)	301 (37.2)	
**HbA1C, n (%**)						<.001
<6.5	1599 (49.6)	525 (64.9)	446 (55.3)	342 (42.5)	286 (35.4)	
≥6.5	1628 (50.4)	284 (35.1)	361 (44.7)	462 (57.5)	521 (64.6)	
FBG (mg/dL) mean ± SD	146.7 ± 60.2	127.2 ± 47.6	140.4 ± 57.0	152.8 ± 58.6	166.4 ± 68.8	<.001
TG (mg/dL) median (IQR)	126.0 (89.0, 187.0)	91.0 (67.0, 122.0)	123.0 (91.0, 177.0)	147.0 (100.0, 208.0)	164.0 (116.0, 238.0)	<.001
BMI (kg/m^2^) mean ± SD	31.5 ± 7.4	24.0 ± 2.6	28.5 ± 2.1	32.6 ± 2.7	40.9 ± 6.5	<.001
**Asthma, n (%**)						<.001
Yes	484 (15.0)	96 (11.9)	92 (11.4)	125 (15.5)	171 (21.1)	
No	2752 (85.0)	713 (88.1)	717 (88.6)	684 (84.5)	638 (78.9)	

ALT = alanine aminotransferase, AST = aspartate aminotransferase, BMI = body mass index, FBG = fasting blood glucose, HDL = high density lipoprotein cholesterol, IQR = interquartile range, LDL = low-density lipoprotein cholesterol, PIR = poverty-income ratio, SD = standard deviation, TC = total cholesterol, TG = triglyceride, TyG-BMI = triglyceride glucose-body mass index.

**Table 2:** Univariate analysis.

Variable	OR (95% CI)	*P*
Age(years)	0.99 (0.98–1)	.005
**Sex, n (%**)		
Female	1 (ref)	
Male	0.71 (0.58–0.86)	<.001
**Race, n (%**)		
Non-Hispanic white	1 (ref)	
Non-Hispanic black	1.1 (0.87–1.4)	.426
Mexican American	0.45 (0.32–0.63)	<.001
Other races	1.07 (0.82–1.38)	.62
**Education level, n (%**)		
<9 years	1 (ref)	
9–12 years	1.22 (0.91–1.64)	.183
>12 years	1.43 (1.08–1.92)	.014
**Marital status, n (%**)		
Living with partner	1 (ref)	
Living alone	1.27 (1.05–1.54)	.016
**PIR, n (%**)		
≤1.3	1 (ref)	
1.3–3.5	0.82 (0.66–1.02)	.077
>3.5	0.61 (0.47–0.8)	<.001
**Smoke, n (%**)		
Never smoking	1 (ref)	
Former smoker	1.4 (1.12–1.75)	.003
Current smoker	1.9 (1.48–2.44)	<.001
**Drinking, n (%**)		
Never drinking	1 (ref)	
Former drinker	1.4 (1.01–1.94)	.045
Current drinker	1.17 (0.9–1.53)	.247
**Hypertension, n (%**)		
No	1 (ref)	
Yes	1.43 (1.18–1.74)	<.001
HDL (mg/dL)	1 (0.99–1)	.483
LDL (mg/dL)	1 (1–1)	.272
TC (mg/dL)	1 (1–1)	.358
ALT (µ/L)	1 (1–1.01)	.55
AST (µ/L)	1 (1–1)	.978
Eosinophil count, median (IQR)	2.85 (1.77–4.61)	<.001
Percentage of eosinophils, median (IQR)	1.08 (1.04–1.12)	<.001
Neutrophil count, mean ± SD	1.07 (1.01–1.12)	.02
Percentage of neutrophil, mean ± SD	1 (0.99–1.01)	.757
**Family history of asthma, n (%**)		
No	1 (ref)	
Yes	2.86 (2.3–3.54)	<.001
**Family history of diabetes, n (%**)		
No	1 (ref)	
Yes	1.4 (1.14–1.71)	.001

ALT = alanine aminotransferase, AST = aspartate aminotransferase, BMI = body mass index, HDL = high density lipoprotein cholesterol, LDL = low-density lipoprotein cholesterol, PIR = poverty-income ratio, SD = standard deviation, TC = total cholesterol.

**Table 3:** Associations of TyG-BMI with asthma in participants with T2D(n = 3236).

	Crude model*		Model 1†		Model 2‡		Model 3§		Model 4∥	
	OR (95 % CI)	*P*	OR (95 % CI)	*P*	OR (95 % CI)	*P*	OR (95 % CI)	*P*	OR (95 % CI)	*P*
Per 10 unit increase	1.04 (1.03–1.06)	<.001	1.04 (1.03–1.05)	<.001	1.04 (1.03–1.05)	<.001	1.04 (1.02–1.05)	<.001	1.04 (1.03–1.06)	<.001
**Quartiles**										
Q1 (<234.705)	1 (Ref)		1 (Ref)		1 (Ref)		1 (Ref)		1 (Ref)	
Q2 (234.705 to < 277.245)	0.95 (0.7–1.29)	.756	1 (0.74–1.36)	.992	1.01 (0.74–1.37)	.972	0.98 (0.72–1.35)	.919	1.03 (0.75–1.43)	.835
Q3 (277.245 to < 328.279)	1.36 (1.02–1.81)	.036	1.42 (1.06–1.89)	.018	1.43 (1.07–1.91)	.017	1.33 (0.99–1.8)	.062	1.41 (1.02–1.94)	.038
Q4 (≥328.279)	1.99 (1.52–2.61)	<.001	1.95 (1.47–2.57)	<.001	1.93 (1.45–2.55)	<.001	1.8 (1.35–2.42)	<.001	1.95 (1.4–2.72)	<.001
*P* for trend		<.001		<.001		<0.001		<.001		<.001

T2D = type 2 diabetes, TyG-BMI = triglyceride glucose-body mass index.

* Crude model: no adjusted.

† Model 1: adjusted for age, gender, and race.

‡ Model 2: adjusted for the variables in Model 1 plus marital status, education level, and PIR.

§ Model 3: adjusted for the variables in Model 2 plus smoking status, drinking status, hypertension, family history of diabetes, family history of asthma.

∥ Model 4: adjusted for the variables in Model 3 plus HDL, LDL, TC, ALT, AST, eosinophil count, percentage of eosinophils, neutrophil count, percentage of neutrophil.

The percentages of missing values were <10%. We imputed missing data of the covariates by using multiple imputations. Five datasets were created and analyzed together. All analyses were performed using the statistical software packages R (http://www.R-project.org, The R Foundation) and Free Statistics software version 2.1.1 (Yantai Zhongzhi Xingyuan Information Technology Co., Ltd., Yantai, Shandong Province, China). Statistical significance was set at *P* < .05.

## 3. Results

### 3.1. Study population characteristics

Our study included 3236 participants (Fig. 1). Table 1 presents baseline characteristics stratified by TyG-BMI quartiles. Significant intergroup differences were observed in age, sex, race, educational level, hypertension status, HDL, LDL, TC, ALT and asthma prevalence (*P* < .05). Participants in the highest TyG-BMI quartile (Q4, ≥328.279) demonstrated distinct demographic patterns-predominantly female (55.4% vs 42.9–51.3% in lower quartiles), younger (mean age 55.6 vs 60.6–62.9 years), and comparable or slightly higher educational attainment (43.1% with >12 years education vs 39.6–41.9% in other groups). This group exhibited adverse metabolic profiles characterized by lower HDL levels (45.0 mg/dL vs 47.2–58.8 mg/dL), elevated TGs (median 164 mg/dL vs 91–147 mg/dL), higher fasting glucose (166.4 mg/dL vs 127.2–152.8 mg/dL), and extreme BMI values (40.9 kg/m² vs 24.0–32.6 kg/m²). Notably, Q4 participants showed disproportionately higher rates of hypertension (59.4% vs 42.7–56%), diabetes family history (62.8% vs 47.6–60.4%), and asthma diagnosis (21.1% vs 11.4–15.5%) compared to lower quartiles.

### 3.2. Association between TyG-BMI and asthma in participants with T2D

In the univariate analysis, male sex, race (Mexican American), higher education levels (>12 years), living alone, smoke (former smoker and current smoker), former drinker, higher PIR (>3.5), hypertension, eosinophil count, percentage of eosinophils, neutrophil count, family history of diabetes and family history of asthma were significantly associated with asthma (Table 2).

Table 3 presents the associations of TyG-BMI with asthma in participants with T2D. ORs (95% CI) of asthma were presented for different quartiles of TyG-BMI. In the crude model, compared to Q1, participants in Q3 (OR = 1.36 [95% CI 1.02–1.81], *P* = .036) and Q4 (OR = 1.99 [95% CI 1.52–2.61], *P* < .001) had significantly increased risks. Significantly, participants in Q4 had a 99% increased risk in the odds of the development of asthma. After adjustment for different covariates in Model 1, Model 2, and Model 3, the odds ratios remained statistically significant. In Model 4, additionally adjusted for all covariates, the ORs for Q3 and Q4 were 1.41 (95% CI 1.02–1.94, *P* = .038) and 1.95 (95% CI 1.40–2.72, *P* < .001), respectively. A significant trend was observed across quartiles in all models (*P* for trend < .001).

**Figure 2.**
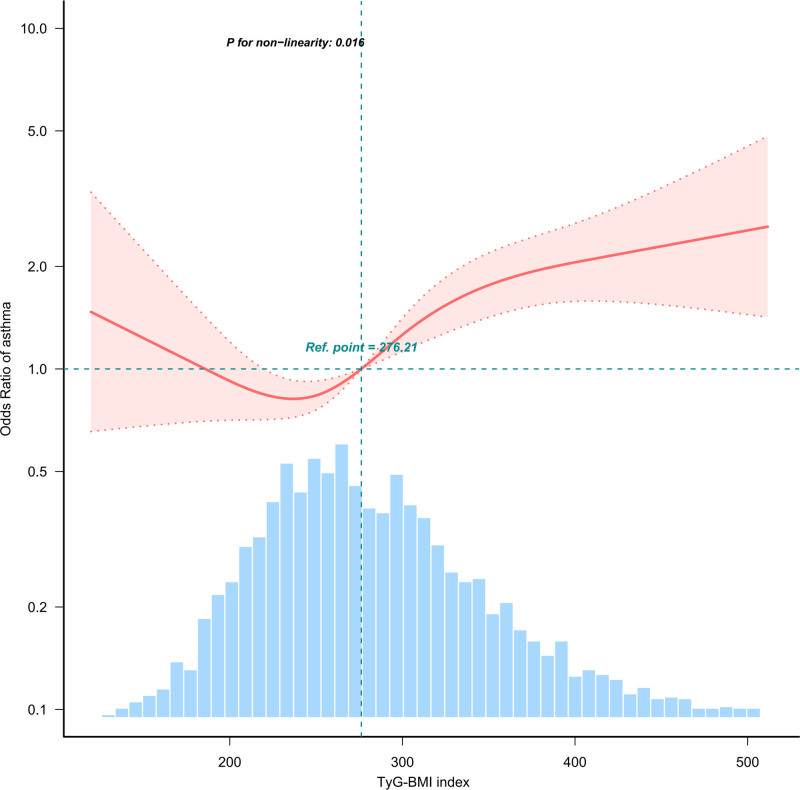
Association between TyG-BMI and asthma odds ratio. Solid and dashed lines represent the predicted value and 95% confidence intervals. Only 99% of the data is shown. TyG-BMI = triglyceride glucose-body mass index.

In the threshold effect analysis, the results showed that TyG-BMI was nonlinearly correlated with asthma prevalence (Fig. 2, *P* for non-linearity = .016), with an inflection point of 208.96, indicating different prevalence levels at different TyG-BMI levels. These findings are detailed in Table 4. Above the inflection point, a 10-unit increase in TyG-BMI was associated with a 5.4% higher odds of asthma.

**Table 4:** Threshold effect analysis for association of per 10-unit TyG-BMI with asthma.

TyG-BMI	Adjusted model	
	OR (95% CI)	*P*
<20.896	0.962 (0.79–1.172)	.7037
≥20.896	1.054 (1.034–1.074)	<.001
Likelihood ratio test		.046

TyG-BMI = triglyceride glucose-body mass index.

**Figure 3.**
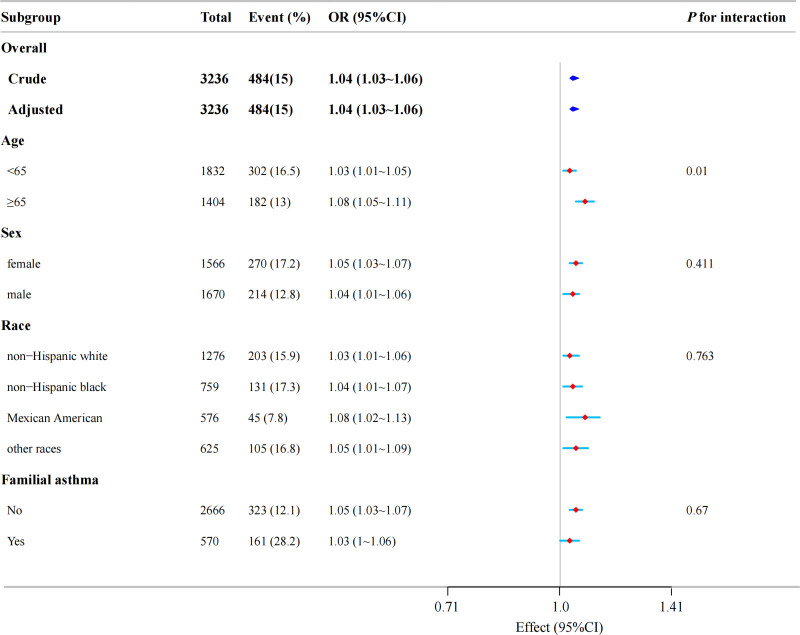
Forest plot of multivariable logistics analysis between TyG-BMI and asthma. TyG-BMI = triglyceride glucose-body mass index.

In addition, in the subgroup analysis stratified by age, sex, race, familial asthma, the association between TyG-BMI and asthma was explored (Fig. 3). The effect size of TyG-BMI on asthma in each subgroup remained stable. The interaction analysis of TyG-BMI with sex, race, familial asthma in regard to asthma were not significant. In the age-stratified analysis, a significant age interaction was observed for the association between per 10-unit increase in TyG-BMI and asthma risk (*P* for interaction = .010). Specifically, the OR was significantly higher in participants aged ≥65 years (1.08, 95% CI 1.05–1.11) compared to those <65 years (1.03, 95% CI 1.01–1.05), suggesting that elderly patients with T2D exhibit greater susceptibility to TyG-BMI-related metabolic disturbances in asthma pathogenesis. This differential association may be attributable to age-related immune-metabolic remodeling, warranting further investigation into the underlying molecular mechanisms.

## 4. Discussion

In this observational study of individuals with T2D in the United States, we found a significant nonlinear relationship between TyG-BMI and asthma risk (*P* for non-linearity = .016). Threshold effect analysis indicated that when TyG-BMI exceeded 208.96, each 10-unit increase was linked to a 5.4% increase in asthma risk (OR = 1.054, 95% CI 1.034–1.074, *P* < .001). The likelihood ratio test yielded a *P*-value of .046, supporting the existence of a threshold effect. These results suggest that TyG-BMI has a significant threshold effect on asthma risk, with higher levels linked to increased risk. This has important implications for asthma prevention strategies in individuals with T2D.

In recent years, the association between IR and asthma in patients with T2D has attracted widespread attention. In an animal study, hyperinsulinemia leads to the inhibition of neuronal M_2_ receptor function, which in turn results in increased acetylcholine release, thereby inducing vagus nerve-mediated airway hyperresponsiveness. This mechanism is independent of inflammation and structural changes in the airways, representing a form of airway hyperresponsiveness caused by neuroregulatory abnormalities.^[12]^ A study had found that in patients with T2D, visceral fat accumulation was significantly associated with IR, and both of these were related to an increased risk of asthma occurrence.^[11]^ At the molecular level, IR may affect the pathogenesis of asthma by regulating the expression of specific genes. For example, insulin has been proven to inhibit the expression of asthma-related genes (such as IL-4, ADAM-33, and LIGHT), thereby possibly reducing the inflammatory response.^[22]^ Furthermore, Insulin resistance-related hyperinsulinemia is linked to α-smooth muscle actin and peribronchial collagen deposition, and higher β-catenin levels. These factors indicate airway smooth muscle cell proliferation and suggest that IR may have irreversible pro-constrictive and profibrotic effects in the lungs.^[23]^ The therapeutic arsenal for asthma may potentially be expanded to include antidiabetic drugs, given their pleiotropic effects. Metformin, the cornerstone of diabetes treatment, alleviates the asthmatic burden by countering IL-6-mediated inflammation, oxidative stress, and insulin resistance. Simultaneously, newer agents such as glucagon-like peptide-1 receptor agonists offer a multifaceted approach by improving insulin sensitivity and glycaemic control while consistently inducing weight loss. This weight-loss effect is clinically significant, as a reduction of 5% to 10% in body weight is a well-documented correlate of enhanced asthma control.^[24–27]^

In addition, the association between obesity and asthma in individuals with T2D is a complex interplay of metabolic and inflammatory pathways. Obesity is a recognized factor in the development of asthma, as evidenced by a study showing that various obesity indicators and IR are associated with wheezing and asthma-like symptoms.^[10]^ The chronic inflammatory state associated with obesity features dysregulated production of multiple inflammatory mediators, including Toll-like receptor 4, tumor necrosis factor-α, and IL-6.^[28]^ Adipokines, secreted by adipose tissue, trigger the innate immune response in adipocytes, leading to sustained inflammation. This adipokine-driven inflammatory milieu can exert adverse effects on systemic cellular and functional organs, including the lungs.^[29]^ These adipokines modulate airway inflammation and bronchial hyperresponsiveness. A Singaporean Chinese adult cohort study prospectively demonstrated that self-reported, physician-diagnosed asthma was associated with an increased risk of type 2 diabetes, an association that was partially explained by BMI and was more pronounced in adults with adult-onset asthma than in those with childhood-onset asthma, as well as in obese participants with asthma compared to nonobese ones.^[30]^ Asthma and obesity may synergistically promote a rise in proinflammatory cytokines within the circulation, consequently heightening the likelihood of IR and T2D. This heightened inflammatory burden likely constitutes a primary etiological factor driving the co-development of both conditions.^[5]^

Our findings contribute to deepening the understanding of the link between IR, obesity, metabolic profile and asthma, particularly in individuals with T2D. In addition to its link with asthma, the specific mechanisms underlying the TyG-BMI’s association with the aforementioned diseases remain to be further explored. While HOMA-IR serves as a traditional indicator of IR, its expensive and complex nature has nonetheless limited its widespread application.^[31]^ The TyG-BMI combines glucose metabolism indicators, lipid metabolism indicators, and anthropometric indicators, which has high diagnostic value in identifying IR.^[32]^

Our research has several advantages. First, our analysis, using multivariable regression, found a positive correlation between TyG-BMI and asthma among individuals with T2D in the USA. Second, the nationally representative sample, diverse racial composition, large sample size, and stable results strengthen our findings. Finally, these findings may offer valuable insights that could inform nutrition education efforts and public health campaigns by relevant institutions and government departments, helping to guide the public toward a healthier lifestyle.

However, our study had some limitations. First, as our data were derived from the NHANES database, the study is cross-sectional. While we found an association between the TyG-BMI and asthma, the cross-sectional nature of the study precludes the determination of causality. Longitudinal studies are needed to explore this relationship further. Second, some diabetes-related data relied on self-reports, which may have introduced recall and reporting biases. Despite these limitations, our findings highlight the need for future multicenter controlled trials to confirm the observed association between the TyG-BMI and asthma.

## 5. Conclusion

This observational study of 3236 participants with T2D reveals a significant nonlinear relationship between the TyG-BMI and asthma risk. The results indicate that each 10-unit increase in TyG-BMI is associated with a 5.4% rise in asthma risk when TyG-BMI exceeds 208.96. This suggests TyG-BMI could be an important indicator for assessing asthma risk in individuals with T2D. The study underscores the importance of metabolic control in asthma prevention and management among patients with T2D.

## Acknowledgments

We are grateful to Dr Jie Liu of the Department of Vascular and Endovascular Surgery, Chinese PLA General Hospital for his contributions to the statistical support and study deign consultations, and our colleagues for their assistance with this study.

## Author contributions

**Conceptualization:** Shengjia Zheng.

**Formal analysis:** Shengjia Zheng.

**Investigation:** Minghe Wang, Yi Fang.

**Methodology:** Minghe Wang, Yi Fang.

**Supervision:** Yuanmei Gao.

**Validation:** Yuanmei Gao.

**Writing – original draft:** Shengjia Zheng.
